# Protocol of the COSMIN study: COnsensus-based Standards for the selection of health Measurement INstruments

**DOI:** 10.1186/1471-2288-6-2

**Published:** 2006-01-24

**Authors:** LB Mokkink, CB Terwee, DL Knol, PW Stratford, J Alonso, DL Patrick, LM Bouter, HCW de Vet

**Affiliations:** 1 Institute for Research in Extramural Medicine (EMGO Institute; www.emgo.nl), VU University Medical Center (VUmc), Amsterdam, The Netherlands; 2 Department of Clinical Epidemiology and Biostatistics, VU University Medical Center (VUmc), Amsterdam, The Netherlands; 3 School of Rehabilitation Science and Department of Clinical Epidemiology and Biostatistics, McMaster University, Hamilton, Canada; 4 Health Services Research Unit, Institute Municipal d'Investigacio Medica (IMIM-IMAS), Barcelona, Spain; 5 Department of Health Services, University of Washington, Seattle, USA

## Abstract

**Background:**

Choosing an adequate measurement instrument depends on the proposed use of the instrument, the concept to be measured, the measurement properties (e.g. internal consistency, reproducibility, content and construct validity, responsiveness, and interpretability), the requirements, the burden for subjects, and costs of the available instruments. As far as measurement properties are concerned, there are no sufficiently specific standards for the evaluation of measurement properties of instruments to measure health status, and also no explicit criteria for what constitutes good measurement properties. In this paper we describe the protocol for the COSMIN study, the objective of which is to develop a checklist that contains COnsensus-based Standards for the selection of health Measurement INstruments, including explicit criteria for satisfying these standards. We will focus on evaluative health related patient-reported outcomes (HR-PROs), i.e. patient-reported health measurement instruments used in a longitudinal design as an outcome measure, excluding health care related PROs, such as satisfaction with care or adherence. The COSMIN standards will be made available in the form of an easily applicable checklist.

**Method:**

An international Delphi study will be performed to reach consensus on which and how measurement properties should be assessed, and on criteria for good measurement properties. Two sources of input will be used for the Delphi study: (1) a systematic review of properties, standards and criteria of measurement properties found in systematic reviews of measurement instruments, and (2) an additional literature search of methodological articles presenting a comprehensive checklist of standards and criteria. The Delphi study will consist of four (written) Delphi rounds, with approximately 30 expert panel members with different backgrounds in clinical medicine, biostatistics, psychology, and epidemiology. The final checklist will subsequently be field-tested by assessing the inter-rater reproducibility of the checklist.

**Discussion:**

Since the study will mainly be anonymous, problems that are commonly encountered in face-to-face group meetings, such as the dominance of certain persons in the communication process, will be avoided. By performing a Delphi study and involving many experts, the likelihood that the checklist will have sufficient credibility to be accepted and implemented will increase.

## Background

Choosing the appropriate health status measurement instrument for a specific purpose is a difficult and time-consuming task. The choice depends on the proposed use of the instrument, the concept to be measured, the readability of the questions, the requirements and costs associated with the use of the instrument, the burden on the subjects, and, last but not least, the measurement properties of the instruments. The measurement properties concern internal consistency, reproducibility, content and construct validity, responsiveness, and interpretability. Kirshner and Guyatt distinguished three kinds of health status measures, i.e. discriminative, predictive and evaluative measures [1]. Not all measurement properties are equally important for each purpose. For example, responsiveness is only important for evaluative measurement instruments.

Although there is consensus that measurement instruments must have good measurement properties, only general guidelines are available, and there are no explicit and comprehensive criteria for what constitutes good measurement properties. Without clear standards the evidence-based selection of measurement instruments is strongly hampered.

Several authors have suggested standards for the development and evaluation of instruments to measure health status. One of the most elaborate lists was proposed by the Scientific Advisory Committee (SAC) of the Medical Outcomes Trust [2,3]. The SAC defined a set of eight key attributes of instruments to measure health status and (health-related) quality of life and standards with which these attributes should be assessed. Most of the standards concern information that authors should provide when reporting on a reproducibility study or a validation study, e.g. a clear description of the methods of data-collection and reporting of specific estimates and standard errors. In addition, they gave some standards, for example for assessing reliability, and some criteria for good measurement properties, such as cut-off points for ICCs. Another list of standards has been compiled by Bombardier and Tugwell, who developed a checklist to compare and evaluate the usefulness of instruments to measure functional status [4]. They propose 12 rules, referring to 6 major issues: comprehensiveness, credibility, accuracy, sensitivity to change, biological sense, and feasibility. Andresen has also defined standards for assessing instruments to measure disability outcomes. Her standards for measurement properties include validity, reliability, and sensitivity to change, as well as statistical methods such as Rasch analysis for assessing the scaling properties [5]. More guidelines for developing or evaluating measurement instruments are given, e.g. by Chassany et al. [6], McDowell and Jenkinson [7], and by country specific organizations such as the American Psychological Association and the Dutch Professional Association of Psychologists.

However, often lacking in these standards are explicit criteria for what constitutes good measurement properties. For example, an intraclass correlation coefficient (ICC) has often been recommended as the most appropriate measure of reliability. There have been some suggestions about what constitutes a minimal ICC for good reliability, but it is unclear whether this concerns the point estimate or the lower limit of the confidence interval, and whether a minimal sample size is required. For the assessment of construct validity it is often recommended that explicit hypotheses about the expected results should be tested [8]. However there are no criteria for how many hypotheses should be defined, how specific these hypotheses should be, the extent to which these hypotheses should be confirmed for good validity, or characteristics, representativity and the size of the sample for a validation study. This may lead to situations in which one is satisfied about the construct validity when instrument A correlates with a similar instrument B in a sample of 20 patients, but with no justification of the expected magnitude of the correlation, or the precision of its estimate. Another problem is that there is a lack of consensus with regard to the method of assessment for some measurement properties. For example, although there is consensus on the importance of responsiveness, there is no consensus on the best way to assess it [9,10].

This has often resulted in a battery of change coefficients being applied to the same data. A possible explanation for this is that often investigators have not conceptualized and declared the anticipated change characteristic (i.e., whether patients in the sample are likely to undergo homogeneous or heterogeneous change) of the sample [11].

With the rapid increase in the number of instruments that are being developed to measure health status there is also an increase in the publication of systematic reviews in which the measurement properties of these instruments are evaluated and compared. These systematic reviews are important tools for the evidence-based selection of instruments. These reviews focus for instance on outcome measures in specific patient groups [12-16], on instruments to measure (general or disease-specific) quality of life in specific patient groups [17-29], on functional disability questionnaires for patients with upper extremity disorders [30-32] and rheumatoid arthritis [33], and on instruments to assess co-morbidity [34]. In most systematic reviews evidence concerning the measurement properties of the instruments is summarized, but only a few authors use explicit and comprehensive criteria to define good measurement properties. Without such explicit criteria, however, it is difficult to decide on the best instrument.

We recently developed a checklist for the evaluation and comparison of the measurement properties of instruments to measure health status [35], which we used in two systematic reviews [21,30]. This checklist was based on the SAC criteria [2,3] and the Bombardier and Tugwell method [4], supplemented with explicit criteria for good measurement properties, typically as defined within our research group [35]. However, other researchers have used different criteria. There is still no consensus with regard to the best criteria.

In this paper we describe the protocol for the COSMIN study. The aim of this study is to develop COnsensus-based Standards for the selection of health Measurement INstruments. Firstly, we will focus on a homogeneous set of measurement instruments, since it is not clear if these standards and criteria can be applied to all sorts of measurement instruments that measure health status. The initial focus of these standards will be on evaluative health related patient-reported outcomes (HR-PROs). We defined evaluative as instruments which are applied to measure HR-PROs in a longitudinal study to assess change over time. With this definition we exclude measurement instruments which are (1) only used as discriminative instruments, (2) only used for predictive purposes, such as diagnostic or screening instruments, or (3) only used as an independent (prognostic) variable, such as a determinant, confounder or effect modifier in a longitudinal study design. PROs include any endpoint derived from patient reports, whether collected in the clinic, in a diary, or by other means, including single-item outcome measures, event logs, symptom reports, formal instruments to measure health-related quality of life (HRQL), health status, adherence, and satisfaction with treatment [36]. By the restriction to health-related PROs, we exclude for example health care related PROs, such a s satisfaction with care and adherence. When these standards and criteria seems applicable to HR-PRO, the next step is to examine if these can be applied on other measurement instruments, such as performance-based instruments or health care related PROs.

Note that one and the same measurement instrument can be used for different purposes, such as discriminative, evaluative and predictive purposes. The COSMIN standards focus on the evaluative application of measurement instruments.

The COSMIN standards will be made available in the form of an easily applicable checklist. This project consists of the preparation by performing a systematic review and a additional literature search, an international Delphi procedure, and field-testing of the resulting checklist.

### Aim of the study

The aim of the COSMIN study is to develop consensus-based standards for the assessment of evaluative HR-PROs, including explicit criteria for good measurement properties. To develop these standards, the following research questions will be addressed:

1. Which measurements properties should be included in the assessment of evaluative HR-PROs, and how should they be defined?

2. How should these measurement properties be assessed in terms of study design and statistical analysis? (i.e. standards)

3. Which criteria should be applied to define what good measurement properties are?

## Method

### The Delphi procedure

The Delphi procedure is basically a series of sequential questionnaires or 'rounds', interspersed by controlled feedback, that seeks to achieve consensus of opinion among a panel of experts [37]. The Delphi procedure is a tool that can be used to generate a debate and to structure and organize a group communication process [37]. It is not a method for creating new knowledge, but rather a process for making the best use of available information [37]. The first round consists of a questionnaire with a large item pool, to identify issues to be addressed in later rounds. An item pool is a set of items regarding all possible issues on a subject. In our study, for example, these issues concern all proposed measurement properties, standards and criteria to judge these with. All panel members are asked to give their opinion about each item, and they also have the opportunity to add additional items. The second and subsequent questionnaires are more specific and aim to converge opinions and to reach consensus.

### Preparation for the Delphi procedure: a systematic review and a additional literature search

To prepare the questionnaires for the Delphi procedure, a systematic literature review will be performed to search for systematic reviews of evaluative health status measurement instruments that describe measurement properties, quality standards and criteria. To be able to find as many as possible standards and criteria we did not yet use the narrow concept of only HR-PROs. Health status measurement instruments include HR-PROs, performance based measures, clinical ratings, etc. We will search in PubMed, Embase and Psycinfo to find systematic reviews of health status measurement instruments. Articles will be included that meet the following inclusion criteria: (1) 'systematic review', (2) the purpose of the review is to find systematically all available 'health status instruments' regarding a specific topic or a specific population, i.e. all questionnaires or performance based measures, etc. or a combination of any of these, (3) the health status instrument has to be applicable as an evaluative measure (i.e. not discriminative or predictive (i.e. diagnostic or screening)), and (4) the purpose of the review is to report on the clinimetric properties of the measurement instruments. Systematic reviews of diagnostic or screening instruments will be excluded. Two of the authors (LM en CT) will perform the selection of articles independently, based on abstracts and if necessary on full text.

An additional literature search in PubMed, Embase and Psycinfo of methodological articles and textbooks presenting comprehensive checklists for standards and criteria will be carried out. The purpose of both the review and the additional literature search is to determine which measurement properties are reported in systematic reviews and in existing standards published in methodological papers and textbooks for the evaluation of health status measurement instruments, which measurement properties should be assessed and how (standards), and which criteria are used to define good measurement properties.

### Design of the Delphi procedure of the COSMIN study

**Figure 1:**
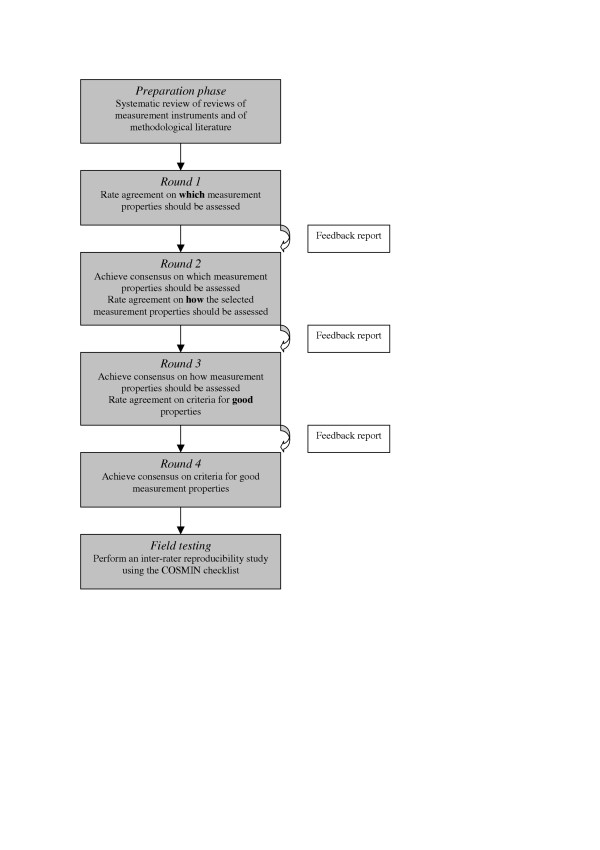
the Delphi procedure of the COSMIN study.

In the Delphi procedure of the COSMIN study, four Delphi rounds are planned, as outlined in Figure 1. Based on the results of the two searches described above, we will developdevelop a pool of all measurement properties, standards and criteria for measurement properties that were found in the literature, and ask for the explicit opinions of the panel members on each of these issues.

The first round will focus on (1) agreement on which measurement properties of HR-PROs should be assessed, and how these measurement properties should be defined. Subsequent rounds will focus on (2) how the selected measurement properties should be assessed (standards), and on (3) defining criteria for good measurement properties.

Each of these three subjects will be at issue in at least two subsequent rounds, in order to offer panel members the opportunity to reconsider and, if appropriate, to change their previous opinion in light of the anonymous responses and considerations of the other panel members. Each subsequent questionnaire contains also a feedback report.

In a Delphi procedure the panel members are carefully selected for their knowledge and interest in a specific field. The panel members will be selected by the Steering Committee (LM, CT, DK, PS, JA, DP, LB and HdV), based on the following inclusion criteria: they should be experts on the development and evaluation of health status measurement instruments, and should have credibility according to the target audience, indicated by authorship of multiple frequently cited publications on (the methodology of) this subject in important journals, such as Quality of Life Research, the Journal of Clinical Epidemiology, Medical Care, and BioMed Central Medical Research Methodology.

Panel members with the following scientific backgrounds will be selected: clinical medicine, biostatistics, psychology (psychometrics), and epidemiology (clinimetrics). They will also be selected to represent important organizations, and to facilitate dissemination and implementation of the checklist, i.e. members of the International Society for Quality of Life (ISOQOL), the Mapi Research Institute, Cochrane PRO methods group, the Patient-Reported Outcomes Group and the European Research Group on Health Outcomes (ERGHO), and editors of important relevant journals, such as Quality of Life Research, and the Journal of Clinical Epidemiology.

Approximately 30 panel members (6–7 per category of scientific background) will be considered appropriate [38]. Based on our previous experience [39,40], we expect approximately that 70% of the invited experts will agree to participate, 80% of the participants will return the first questionnaire, and 65% of these will also return the second and subsequent questionnaires. Therefore, we will initially invite 80 experts to participate (20 per category of scientific background). Those who are invited will be asked to inform us whether or not they wish to participate. If less than 55 are willing to participate, more persons will be invited, until 55 have agreed to participate. If one of the categories of scientific background is under-represented, additional experts will be invited from that category. The identity of the panel members will be kept unknown to the other panel members until all rounds has been completed. Furthermore, the responses will be distributed anonymously among to the panel members and the Steering Committee (except for the member of the Steering Committee who is responsible for the correspondence).

### Consensus

To reach consensus on each of the three issues outlined above, the same strategy will be carried out in each round. In the first questionnaire the panel members will be asked to rate how strongly they (dis)agree to include each measurement property in the checklist (e.g. to which extent do you agree that internal consistency should be included in the assessment of evaluative HR-PROs?), and what the most appropriate definition for each measurement property is. Ratings will be scored on a 5-point Likert scale (ranging from strongly agree to strongly disagree). The panel members will be asked to give considerations and arguments to support their opinion. They will also be given the opportunity to suggest alternative wordings, to suggest additional measurement properties, or to make any other comments.

In the second questionnaire the aim is to reach consensus on the items included in the first questionnaire, i.e. to reach consensus on which relevant measurement properties should be included in the COSMIN checklist, and on their definition. An anonymous feedback report of the results of the first round will be distributed among the panel members with the second questionnaire. The results of Delphi round 1 will be presented both quantitatively (the distribution and mean (or median) scores on the 5-point Likert scales) and qualitatively (the suggestions and comments of the panel members concerning each measurement property and the definitions). The panel members will again be asked to give their opinion on each item. In principle, only measurement properties for which minimal 67% scored at least 3 points in the second round will be selected for inclusion, but the Steering Committee has the right to make alternative decisions after reviewing the responses. If different terminology is proposed by certain panel members, the Steering Committee will choose one term, and will provide a description of synonyms. The decisions made by the Steering Committee will be presented and justified in the feedback report between the Delphi rounds. The panel members will be given the opportunity to react to, and (dis)approve these decisions.

This procedure will be repeated for each issue, i.e. which measurement properties should be assessed, how these properties should be assessed, and which criteria should be used to define what good measurement properties.

The Steering Committee will decide whether or not consensus was reached. In general, consensus will be defined as "a general agreement among a substantial majority (i.e. 67% had a score of at least 3 points on the 5-point Likert scale) of panel members". It is expected that it will be possible to reach consensus on which measurement properties should be assessed and how they should be assessed. However, we anticipate that it will be much more difficult to reach consensus on the criteria for good measurement properties, because this often depends on the situation in which the criteria will be applied. A possible outcome of our study will therefore be a checklist with consensus-based standards for the evaluation of the measurement properties of HR-PROs, but with no explicit criteria for good measurement properties for all the measurement properties.

### Field-testing

After the four Delphi rounds, a first version of the checklist and a user's manual will be prepared by the Steering Committee. This checklist will be tested in a inter-rater reproducibility study, in which a number of raters will be asked to judge a selection of validation studies of a variety of measurement instruments. Inter-rater variability will be determined on each item of the checklist.

## Discussion

The Delphi procedure is particularly suitable for the development of consensus-based standards for the evaluation of HR-PROs for several reasons:

- A Delphi approach is especially useful for situations in which there is a lack of empirical evidence and decisive factors are rather subjective, and not knowledge based.

- It is useful for situations in which strong differences of opinion, e.g. due to differences in expertise and scientific backgrounds, as is anticipated in this study.

- The checklist can be developed in co-operation with many experts in the field of health status measurement from different scientific backgrounds and from different countries. By providing feedback from previous rounds, the Delphi technique provides the advantage of a group process of building on the work and expertise of all panel members.

- The Delphi technique avoids problems that are commonly encountered in face-to-face group meetings, such as the dominance of certain persons in the communication process, and the geographical constraints and expenses of bringing together a group of experts. The panel members can be kept unaware of the identity and opinions of the other panel members, which allows them to express their personal views freely.

- Checklists developed by individual experts or small research groups from one institute, such as our checklist [35], do not have sufficient credibility to be accepted and implemented. Only checklists developed in international collaborations, such as the OMERACT initiative [41], will have a fair chance of becoming widely used.

### Final remark

The purpose of the COSMIN study is to develop consensus-based standards to assess the quality of evaluative HR-PROs, so that in the future this can be assessed more uniformly. The checklist (containing these standards) can be used by researchers, reviewers of journals or professionals for the development, evaluation and selection of measurement instruments, the planning of validation studies, and the critical appraisal of them. Our standards should contribute to the improvement of the quality of (validation studies of) HR-PROs.

We expect the final version of the checklist to be ready in 2008/2009.

## Competing interests

The author(s) declare that they have no competing interest.

## Authors' contributions

LM is the principal investigator of the study described in this article. CT, DK, LB and HdV developed the initial study protocol. All authors participated in the design and preparation of the study. LM wrote the first draft of the manuscript. All other authors commented on this draft and contributed to the final manuscript.

## Pre-publication history

The pre-publication history for this paper can be accessed here:


